# Biochar, activated carbon, and carbon nanotubes have different effects on fate of ^14^C-catechol and microbial community in soil

**DOI:** 10.1038/srep16000

**Published:** 2015-10-30

**Authors:** Jun Shan, Rong Ji, Yongjie Yu, Zubin Xie, Xiaoyuan Yan

**Affiliations:** 1 State Key Laboratory of Soil and Sustainable Agriculture, Institute of Soil Science, Chinese Academy of Sciences, Nanjing 210008, China; 2 Changshu Agro-ecological Experimental Station, Chinese Academy of Sciences, Changshu 215555, China; 3 State Key Laboratory of Pollution Control and Resource Reuse, School of the Environment, Nanjing University, 163 Xianlin Avenue, Nanjing 210023, China; 4 College of Applied Meteorology, Nanjing University of Information Science and Technology, Nanjing 210044, China

## Abstract

This study investigated the effects of biochar, activated carbon (AC)-, and single-walled and multi-walled carbon nanotubes (SWCNTs and MWCNTs) in various concentrations (0, 0.2, 20, and 2,000 mg/kg dry soil) on the fate of ^14^C-catechol and microbial community in soil. The results showed that biochar had no effect on the mineralization of ^14^C-catechol, whereas AC at all amendment rates and SWCNTs at 2,000 mg/kg significantly reduced mineralization. Particularly, MWCNTs at 0.2 mg/kg significantly stimulated mineralization compared with the control soil. The inhibitory effects of AC and SWCNTs on the mineralization were attributed to the inhibited soil microbial activities and the shifts in microbial communities, as suggested by the reduced microbial biomass C and the separated phylogenetic distance. In contrast, the stimulatory effects of MWCNTs on the mineralization were attributed to the selective stimulation of specific catechol-degraders by MWCNTs at 0.2 mg/kg. Only MWCNTs amendments and AC at 2,000 mg/kg significantly changed the distribution of ^14^C residues within the fractions of humic substances. Our findings suggest biochar, AC, SWCNTs and MWCNTs have different effects on the fate of ^14^C-catechol and microbial community in soil.

Naturally occurring phenols, which are pervasive precursors of soil humic substances, play a crucial role in the transformation and stabilization of soil carbon and nitrogen^1^,^2^. They account for up to 10% of the total dissolved organic carbon in soil^3^ and are primarily the product of the degradation of biopolymer lignin, microbial synthesis, and plant root exudation^4^,^5^. Naturally occurring phenols often undergo oxidative coupling reactions in soil under the catalysis of oxidoreductive enzymes (e.g., peroxidase and laccase) and metal oxides (e.g., Fe and Mn oxides), resulting in their polymerization with humic substances^6^,^7^,^8^,^9^. In addition, phenolic compounds are of great importance in determining the stability of organic matter in soil, as they possess antioxidant activity^10^.

Carbonaceous materials, such as biochar, activated carbon (AC), and carbon nanomaterials [e.g., single-walled and multi-walled carbon nanotubes (SWCNTs and MWCNTs)], have been the subject of many research efforts due to their unique physico-chemical characteristics (e.g., large surface area, high microporosity, and superb sorption capacities), increased occurrence in the environment and potential value in remedying contaminated soil and sediments^11^,^12^,^13^,^14^,^15^. The application of these carbonaceous materials to soil can effectively alter the bioavailability and bioaccessibility of organic compounds (including organic contaminants and naturally occurring phenols), and hence their uptake by plants and earthworms^11^,^14^,^16^,^17^,^18^,^19^,^20^,^21^,^22^. The effects of these carbonaceous materials in soil on the sorption and degradation of organic contaminants has been widely studied in the past decades^11^,^13^,^14^,^16^; however, relatively little is known about the effects of these carbonaceous materials on the mineralization and transformation of naturally occurring phenols in soil^22^.

Catechol is a basic constituent of many naturally occurring phenols and regarded as an important precursor of humic substances^23^ enabling it as a representative of naturally occurring phenols. Using ^14^C tracer, we examined the effects of biochar, AC, and carbon nanotubes (SWCNTs and MWCNTs) at a log scale of concentrations (0, 0.2, 20, and 2,000 mg/kg dry soil) on the mineralization, transformation and residue distribution of ^14^C-catechol in an agricultural soil. The soil bacterial communities and compositions as affected by these carbonaceous materials were also profiled using 454 pyrosequencing of 16 S rRNA genes. We used uniformly ^14^C-labeled catechol to facilitate the localization of the fate of catechol.

## Results

### Adsorption and desorption of ^14^C-catechol on soil and carbonaceous materials

**Figure 1:**
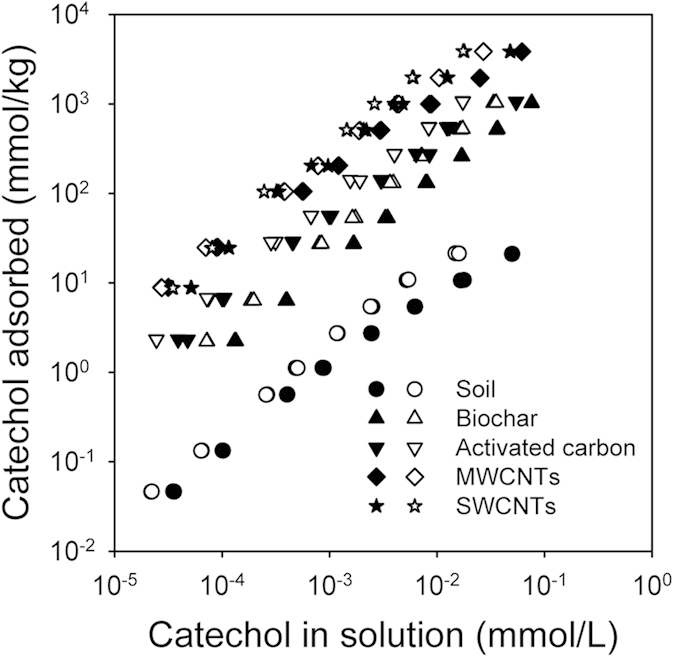
Adsorption and desorption isotherms of ^14^ C-catechol on soil and various carbonaceous materials (Biochar, Activated carbon, SWCNTs, and MWCNTs).

Adsorption and desorption isotherms of ^14^C-catechol on soil and carbonaceous materials are presented in Fig. 1. The Freundlich model fits the isotherm data (*R*^2^ ≥ 0.98) well (Table 1). Based on the *K*_F_ and *K*_d_ values, the adsorption of ^14^C-catechol on the carbonaceous materials was considerably higher than that on the soil, with *K*_F_ and *K*_d_ increasing as follows: SWCNTs > MWCNTs > AC > biochar > soil (Table 1). In contrast, the normalized surface area *K*_F_ and *K*_d_ values for the carbonaceous materials were as follows: biochar > AC > MWCNTs > SWCNTs (Table 1; Table S1). All isotherms exhibited non-linearity as the Freundlich *n* values of the soil and carbonaceous materials varied from 0.59 to 0.92 (Table 1).

In all cases, the *K*_F_ and *K*_d_ values of the desorption isotherms were higher than those of the adsorption isotherms (Table 1), implying a partial desorption hysteresis of ^14^C-catechol on the soil and the carbonaceous materials.

### Mineralization of ^14^C-catechol in soil

**Figure 2:**
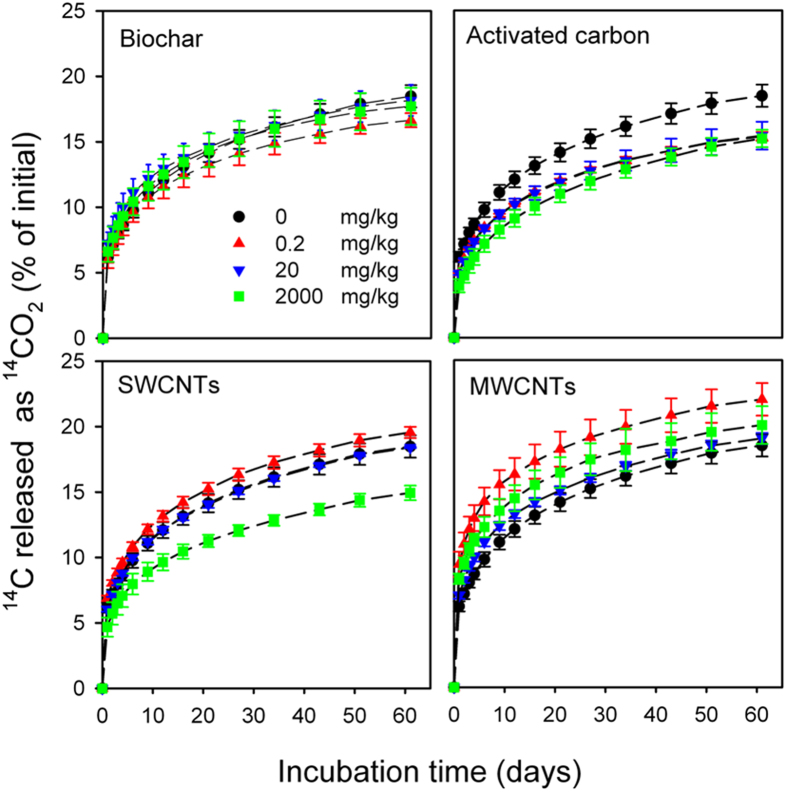
Cumulative release of ^14^ CO_2_ from ^14^ C-catechol in soil without carbonaceous materials (0 mg/kg), and in soil with various concentrations (0.2, 20, and 2,000 mg/kg) of carbonaceous materials during 61 days of incubation at 25 °C. The values are means with standard deviation (*n* = 3).

The mineralization rate of ^14^C-catechol was initially high (days 0–6), decreased progressively until the end of the experiment (Fig. 2) and did not exhibit a lag phase, indicating that the indigenous microorganisms in the soil were capable of mineralizing catechol without an adaptation time. The effects of the carbonaceous materials on the mineralization of ^14^C-catechol depended on the type and addition rate of the carbonaceous material (Type × Addition rate interaction: *F* = 5.8, *P* < 0.001). Compared to the control soil, added biochar had no effect on the mineralization of ^14^C-catechol (*F* = 1.4, *P* = 0.29). In contrast, AC significantly reduced the mineralization of ^14^C-catechol (*F* = 13.0, *P* < 0.001). No significant effect on mineralization was observed for SWCNTs at <20 mg/kg; however, SWCNTs at 2,000 mg/kg significantly (*P* < 0.05) reduced the mineralization of ^14^C-catechol relative to the control soil (Fig. 2). Significantly more (*P* < 0.05) ^14^C-catechol was mineralized when MWCNTs were applied at 0.2 mg/kg than in the control soil, whereas MWCNTs at concentrations of 20 and 2,000 mg/kg did not affect the mineralization of ^14^C-catechol in the soil (Fig. 2).

### Distribution of ^14^C residues in soil

The incorporation of ^14^C-catechol derived residues into dissolved organic matter (DOM), the ^14^C residues distribution among various humic substances fractions and the total radioactivity recovery are summarized in Table 2. The total radioactivity recovery ranged from 90.9% to 95.5%, with an average of 93.8%, confirming that the extraction and determination procedures used in this study were sufficient. The incorporation of ^14^C into DOM in the control soil was negligibly low (<0.3%) and was only significantly affected by SWCNTs at 2,000 mg/kg (0.7%) (Table 2). The effects of carbonaceous materials on the distribution of ^14^C residues among the various humic substances fractions depended on the type and addition rate of the carbonaceous materials (Type × Addition rate interaction: *F* = 2.6, *P* = 0.015). Compared to the control soil, biochar and SWCNTs at all cases had no effect on the amounts of ^14^C residues within the various humic substances fractions, whereas AC at 2,000 mg/kg significantly (*P* < 0.05) increased the amount of ^14^C in the insoluble humin fraction (Table 2). Compared to that in the control soil, MWCNTs significantly (*P* < 0.05) reduced ^14^C amount in the soluble humin fraction at all concentrations, and significantly (*P* < 0.05) reduced the amount of ^14^C in the fulvic acids (FA) and humic acids (HA) fractions only at 2,000 mg/kg (Table 2).

### Molecular size distribution of ^14^C residues with alkaline extractable humic substances

The ^14^C- residues in the alkaline extractable humic substances had one molecular domain with a dominant molecular weight of 580 Da (Fig. S1). The addition of biochar or SWCNTs did not affect this molecular size distribution of the ^14^C residues (Fig. S1), whereas AC at >20 mg/kg and MWCNTs at 2,000 mg/kg shifted slightly the dominant molecules of the ^14^C residues toward the humic substances with higher molecular weights (Fig. S1).

### Effects of carbonaceous materials on the soil microbial biomass C

**Figure 3:**
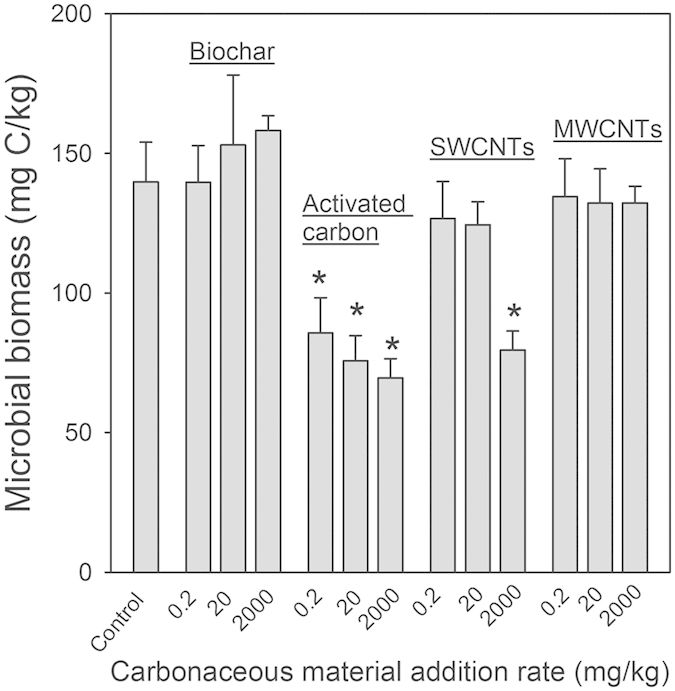
Effects of carbonaceous materials (Biochar, Activated carbon, SWCNTs and MWCNTs) on the soil microbial biomass C at various amendment concentrations (0, 0.2, 20 and 2,000 mg/kg) after 61 days of incubation. The values are means with standard deviation (*n* = 3). Asterisks above the mean values indicate significant differences from those for the control soil.

Soil microbial biomass C contents in carbonaceous material-free control soils and soils amended with various amounts of carbonaceous material are shown in Fig. 3. After 61 days of incubation, biochar and MWCNTs amendments had no effect on the soil microbial biomass C, while AC at 0.2, 20, and 2,000 mg/kg significantly (*P* < 0.05) decreased the soil microbial biomass C by 38.7%, 45.8%, and 50.4%, respectively (Fig. 3). No significant effect on the soil microbial biomass C was observed for SWCNTs at <20 mg/kg; however, soil microbial biomass C significantly (*P* < 0.05) decreased by 43.1% when SWCNTs were applied at 2,000 mg/kg (Fig. 3).

### Effects of carbonaceous materials on the soil microbial community and composition

**Figure 4:**
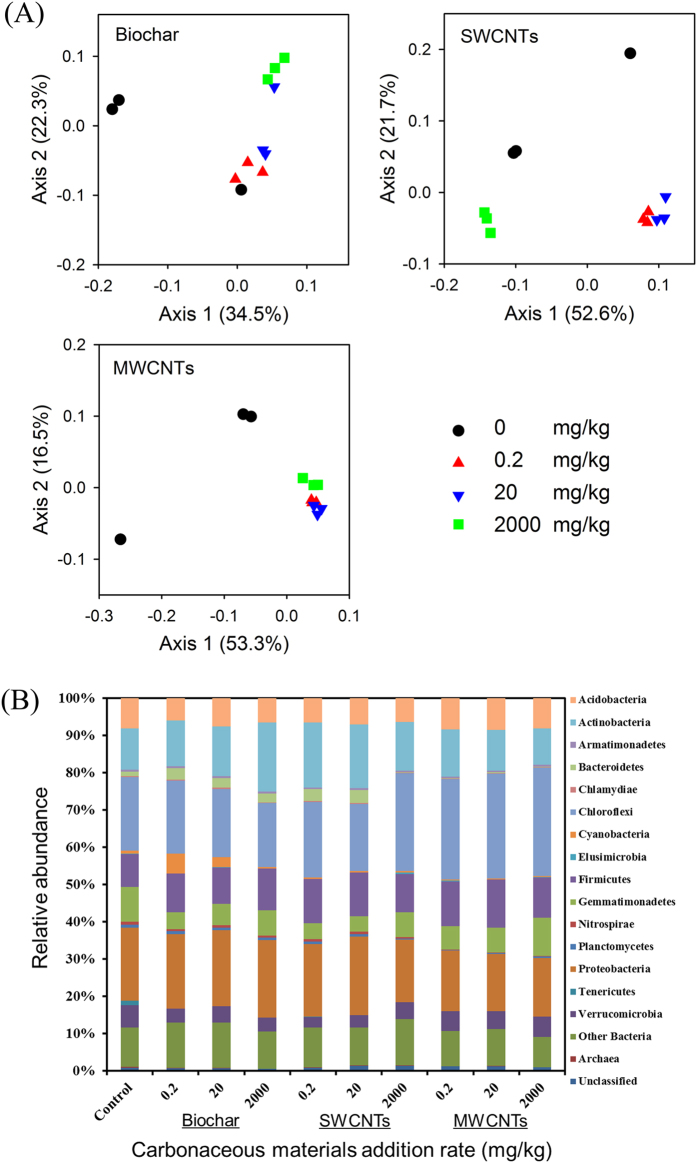
Principal coordinates analysis (PCoA) illustrating the shifts in the soil bacterial communities based on the Bray-Curtis distance (A) and relative abundance of dominant phyla (B) as affected by the presence of carbonaceous materials (Biochar, SWCNTs and MWCNTs) at various amendment concentrations (0, 0.2, 20 and 2,000 mg/kg) after 61 days of incubation.

Changes in the bacterial communities in response to carbonaceous materials amendment were illustrated using principal coordinate analysis (PCoA), which shows that the microbial community structures were shifted after exposure to carbonaceous materials in all cases, except for the 0.2 mg/kg biochar treatment (Fig. 4A). In the case of biochar, the bacterial communities of the 2000 mg/kg treatment were significantly different from those in the 0.2 mg/kg and control treatments (Fig. 4A). In the case of SWCNTs, the 2000 mg/kg treatment was clearly separated from the control, 0.2 and 20 mg/kg treatments, showing that the 2000 mg/kg treatment exerted a different stress to indigenous microbes. In all MWCNTs treatments, the community compositions were obviously different from those in the control treatment (Fig. 4A).

The community changes at the phylum level showed that the bacteria displayed different variation among different groups. Phylogenetic analysis indicated that Proteobacteria, Chloroflexi, Actinobacteria and Firmicutes were the most dominant groups in the microbial community of soil (Fig. 4B). Compared with those in the control treatment, the relative abundances of Tenericutes and Nitrospirae were significantly (*P* < 0.05) reduced in all of the carbonaceous materials amendment treatments (Fig. S2). The occurrence of Verrucomicrobia was significantly (*P* < 0.05) reduced in all of the biochar amendment treatments, being opposite to that of Bacteroidetes (Fig. S2). The occurrence of Actinobacteria was only significantly (*P* < 0.05) decreased by the 2000 mg/kg biochar amendment. For SWCNTs treatments, the relative abundances of Verrucomicrobia, Cyanobacteria, and Gemmatimonadetes were significantly (*P* < 0.05) decreased compared to those for the control (Fig. S2). The abundances of Bacteroidetes and Elusimicrobia were significantly (*P* < 0.05) increased in the 0.2 and 20 mg/kg SWCNTs treatments, but significantly (*P* < 0.05) decreased in the 2000 mg/kg SWCNTs treatment relative to those for the control treatment (Fig. S2). In all of the MWCNTs treatments, the relative abundance of Bacteroidetes was significantly (*P* < 0.05) decreased, whereas, that of Chloroflexi was significantly (*P* < 0.05) increased, in comparison with those of the control (Fig. S2). The abundance of Firmicutes was significantly increased by the 0.2 and 20 mg/kg MWCNTs treatments (Fig. S2).

## Discussion

The adsorption of ^14^C-catechol on biochar, AC, and carbon nanotubes was considerably higher than that on the soil (Fig. 1), indicating that amendments of soil with these carbonaceous materials may increase the ^14^C-catechol sorption affinity to the soils. This adsorption behavior could be described well by the Freundlich model (Fig. 1) and was consistent with that of previous studies of catechol sorption on these carbonaceous materials^19^,^20^,^24^, whereas a higher linearity index (0.59-0.92) was observed in our study than in previous studies.

The desorption hysteresis of ^14^C-catechol on soil and carbonaceous materials was observed in this study, indicating that the adsorption of catechol was partially reversible. The desorption hysteresis on the carbonaceous materials signifies that carbonaceous materials may serve as ^14^C-catechol sinks (e.g., only the reversible portion of ^14^C-catechol could be released) after their addition to soil. Due to the strong dipole moment of catechol, the desorption hysteresis on soil can be attributed to chemical bonding (chemisorption) with soil organic matter (SOM)^25^, strong electrostatic interaction with exchangeable cations in soil, entrapment with condensed organic matter^26^, and the π-π forces between catechol and SOM surface^27^. In contrast, the desorption hysteresis on carbonaceous materials may be caused by the electrostatic and strong π-π interactions of the benzene ring of catechol to the surface of the carbonaceous materials, as well as capillary condensation^27^,^28^.

The influence of carbonaceous materials on the mineralization and transformation of organic compounds in soil may occur in two ways: i) by changing indigenous microbial activities; and ii) by reducing the bioavailability of compounds as a result of their high adsorption affinity for carbonaceous materials^18^,^29^,^30^. The soil microbial biomass C was significantly decreased in the presence of AC at >0.2 mg/kg and SWCNTs at 2,000 mg/kg, indicating that the indigenous microbial growth was inhibited (Fig. 3). This inhibited soil microbial activities contributed to the reduced ^14^C-catechol mineralization. And the significant shifted microbial community structures as observed in the SWCNTs treatments may also play a role in reducing ^14^C-catechol mineralization (Fig. 4A). Consistent with our results, it has been reported that SWCNTs at >300 mg/kg, both in the form of a powder and a suspension, could significantly restrain the activities of most soil enzymes and reduce the microbial biomass C and N^31^. Inhibited microbial growth and microbial activity in the presence of AC and SWCNTs could be attributed to the altered soil physico-chemical processes induced by AC and SWCNT amendments, such as the sorption of inorganic and organic compounds (including enzymes), changes in soil water retention, and pore structure^32^.

The decreased mineralization of ^14^C-catechol by AC and SWCNTs at 2,000 mg/kg could also be a result of the reduced bioavailability in soil amended with AC and SWCNTs because the adsorption of ^14^C-catechol on the AC and SWCNTs was considerably higher than that on the soil, and the adsorption was only partially reversible (Fig. 1). Many studies have demonstrated that AC and SWCNTs amendments can considerably reduce the bioavailability and bioaccessibility of hydrophobic organic pollutants by acting as strong adsorbents, thereby resulting in reduced mineralization and dissipation of hydrophobic organic pollutants in the soil and sediments^14^,^29^,^33^,^34^,^35^.

In contrast to the AC treatment, biochar amendments did not significantly affect ^14^C-catechol mineralization (Fig. 2; Table 2) even though the adsorption of catechol on biochar was only slightly lower than that of AC. Typically, only free or readily desorbed compounds can be accessed and degraded by soil microorganisms; however, recent studies have shown that soil microorganisms may also directly degrade compounds even when they have adsorbed onto the surfaces of black carbon (e.g., charcoal)^36^,^37^. Soil microbial biomass C was not affected by biochar amendments, reflecting that the growth of indigenous microorganisms was probably not constrained in the biochar-amended soil (Fig. 3).

No significant difference was observed between MWCNTs at >20 mg/kg and the control soil for ^14^C-catchol mineralization in the soil (Fig. 2). Thus, MWCNTs at these concentrations did not significantly influence the activity of catechol-degrading microorganisms, and the bioaccessibility of catechol to the catechol-degrader was also unaffected by the presence of MWCNTs, even though the MWCNTs had a considerably higher sorption capacity for catechol relative to the soil. The effects of MWCNT amendments on the microbial biomass C support this speculation, as MWCNTs amendments did not affect the microbial biomass C compared to that of the control soil (Fig. 3). In accordance with our results, it was shown that MWCNTs amendments up to 1,000 mg/kg had no effect on soil respiration, enzymatic activities, and microbial community composition^38^. Microbial activity was also not affected by MWCNTs at a 5% addition rate in sediments amended with a specific bacterial degrader *Agrobacterium,* leading to the rapidly occurring mineralization of ^14^C-phenanthrene^37^. Additionally, MWCNTs have a high sorption affinity to dissolved organic matter (e.g., humic acids, peptone and α-phenylalanine)^39^. The number of sorption sites on the MWCNTs surface available to catechol should be considerably reduced by the sorption of dissolved soil organic matter to MWCNTs, resulting in that the bioaccessibility of catechol in the soil was not significantly affected by the presence of MWCNTs. Surprisingly, MWCNTs at 0.2 mg/kg significantly stimulated the mineralization of ^14^C-catechol in the soil (Fig. 2; Table 2). The reasons for this stimulation by MWCNTs at 0.2 mg/kg might be owing to the alternation of the soil microbial community structures, which were sensitive to MWCNTs at all amendment rates (Fig. 4A). Recent studies have shown that the abundance of the bacterial genera Bacteroidetes, Firmicutes, Rhodococcus, Cellulomonas, Nocardioides and Pseudomonas, which are considered potential degraders of recalcitrant contaminants, increased in the presence of MWCNTs^38^,^40^. In the present study, the relative abundances of Firmicutes were also significantly increased for the 0.2 and 20 mg/kg MWCNTs treatments (Fig. S2).

As the adsorption of ^14^C-catechol on the SWCNTs and MWCNTs was approximately identical, the different effects of SWCNTs and MWCNTs on the mineralization of ^14^C-catechol were mainly attributed to their different effects on the soil microbial community structures and activities. SWCNTs were more toxic to cells than MWCNTs even though both SWCNTs and MWCNTs can penetrate into the cytoplasm and nuclear membranes of the cells, resulting in an increase in cell death^41^. SWCNTs were also shown to be more effective in suppressing enzyme activities and microbial biomass C and N due to the higher surface area of the SWCNTs than the MWCNTs^31^.

The majority of the radioactivity of ^14^C residues (>70%) at the end of the incubation remained in the soil humic substances fractions (Table 2); among these the humin fractions (i.e., the sum of the soluble, and insoluble humin fractions) were predominant followed by the FA and HA fractions. The humin fractions differ from the FA and HA fractions in terms of their C and O content and the quantity of functional groups^42^. Humin fractions are more lipophilic and contain organoclay complexes with high surface area^43^, which may provide more adsorption and incorporation sites for ^14^C residues and facilitate the incorporation of ^14^C residues into the humin fractions. The incorporation of ^14^C residues into the humin indicates the stabilization of ^14^C residues because humin is recalcitrant and represents the stable stage of SOM^42^,^44^. The stabilization of ^14^C residues in soil in this study, which occurred through the binding of ^14^C residues to soil organic and inorganic components, aligned with the results of previous studies, in which more than 70% of ^14^C-catechol derived residues were incorporated into the humin fractions^22^,^45^.

Among the tested carbonaceous materials, MWCNTs had the greatest impact on the distribution of ^14^C residues within the humic substances fractions, with 2,000 mg/kg of MWCNTs significantly decreasing the ^14^C residues in the FA, HA and soluble humin fractions. AC amendments had the next greatest impact, with ^14^C residues significantly increasing in the insoluble humin fractions in the presence of 2,000 mg/kg of AC relative to the levels in the control soil (Table 2). The effects of MWCNTs and AC amendments at 2,000 mg/kg may be reflected in the changes in the molecular size distribution of the ^14^C residues. In the presence of MWCNTs and AC amendments (2,000 mg/kg), the molecular size of the ^14^C residues shifted toward a higher molecular mass within the humic substances (Fig. S1).

Generally, naturally occurring phenols are easily subjected to biotic and abiotic transformations in soil, resulting in the polymerization of their phenolic structure into humic substances via covalent bonding^46^. Because the soil organic (humic substances) and inorganic components (e.g., metal oxides and clay minerals) have large interaction affinities for ^14^C-catechol^4^,^6^,^7^, the biodegradation and transformation of ^14^C-catechol can be determined by its interaction with the soil components rather than by the presence of carbonaceous materials. This may explain why the biochar and SWCNTs amendments had no effect on the ^14^C residues within the various humic substances fractions (Fig. S1; Table 2). These results indicated that carbonaceous material amendments played a minor role in the stabilization process of ^14^C-catechol derived residues in soil.

Altogether, our results suggest that different carbonaceous materials may have different effects on fate of ^14^C-catechol in soil and soil microbial diversity. Biochar has less effect on the fate of ^14^C-catechol in soil than AC and CNTs. As the potential release of biochar, AC, and carbon nanotubes into the environment is increasing with their increasing application, our findings have important implications for an understanding of the fate of ^14^C-catechol in the presence of these carbonaceous materials. Because the predicted average concentration of CNTs in soil (0.01–0.02 μg/kg) is far below those used in the present study^47^, the effects of CNTs (0.2–2,000 mg/kg) on mineralization and transformation of ^14^C-catechol may be significant and relevant in “hot-spot” areas of CNTs-contaminated soils. Nevertheless, these results provide useful information for a worst case scenario when evaluating potential risks and effects of CNTs in the soil. Further studies should focus on the role of specific catechol degraders in the mineralization of ^14^C-catehcol by pure culture-dependent assessment and functional gene analysis.

## Methods

### Soil, carbonaceous materials and chemicals

A pristine soil was collected from an agricultural field (5–15 cm depth) outside of the city of Rudong in Jiangsu Province, China. The fresh soil samples were sieved to less than 2 mm and divided into two portions: one portion was air-dried for a chemical property analysis and adsorption experiment, and the other portion was used for an incubation experiment. The soil had a pH of 6.8 (0.01 M CaCl_2_) and a total organic carbon content of 0.9%. Its sand, silt, and clay contents were 12.9%, 76.1%, and 11.0%, respectively.

The sawdust of *Cunninghamia lanceolata* was used to produce biochar in a muffle furnace under oxygen-limited conditions. The starting pyrolysis temperature was set at 400 °C, which was held constant for 4 h. The resulting biochar was cooled inside the furnace to room temperature. Analytical-grade granular AC was purchased from Huangkang Chemical Co., Ltd. (Shanghai, China). Prior to the experiments, the biochar and AC were ground mechanically and sieved to less than 1 mm. The carbon nanotubes (SWCNTs and MWCNTs) were purchased from Shenzhen Nanotech Port Co. Ltd. (Shenzhen, Guangdong Province, China). The outer diameters of the SWCNTs and MWCNTs were <2 nm and 10–20 nm, respectively. The specific surface area and pore size distribution of the biochar, AC, SWCNTs, and MWCNTs were evaluated using the Brunauer–Emmett–Teller (BET) nitrogen adsorption technique at 77 K. The elemental abundances of the biochar, AC, SWCNTs, and MWCNTs were determined by an elemental analyzer (Elementar Vario EL III, Germany). The characteristics of these carbonaceous materials are summarized in Table S1.

Uniformly ^14^C-labeled catechol (^14^C-catechol) with >99% radiochemical purity was purchased from American Radiolabeled Chemicals (Saint Louis, MO, USA) with a specific radioactivity of 2.82 GBq/mmol. Non-labeled catechol (>99% purity) was purchased from Sigma Incorporation (Shanghai, China).

### Adsorption experiments

Adsorption experiments were performed in glass vials with Teflon-lined screw caps containing certain amounts of adsorbents (1,000 mg for soil, 20 mg for biochar and AC, and 5 mg for SWCNTs and MWCNTs, respectively). Twenty-two milliliters of 0.1–100 mg/L ^14^C-catechol solution prepared in 0.01 M CaCl_2_ and containing 200 mg/L NaN_3_ was added to the vials. The vials were then gently shaken on a vertical rotary shaker at 25 °C in the dark for five days. Our preliminary experiments indicated that five days was a sufficient duration to reach the adsorption equilibrium. After being shaken, the vials were centrifuged at 2,500 g for 15 min, and 0.5 mL of the supernatant was sampled for the determination of ^14^C-catechol using a liquid scintillation counter (LSC). Control experiments containing no adsorbents in the vials showed that the loss of ^14^C-catechol was negligible during the adsorption experiments. All of the adsorption experiments were performed in duplicate.

Desorption experiments were performed immediately after the adsorption experiments by replacing 90% of the supernatant with a 0.01 M CaCl_2_ solution containing 200 mg of NaN_3_. All of the vials were shaken for five days at 25 °C in the dark and centrifuged at 2,500 g for 15 min. The ^14^C-catechol in the supernatant was determined again using LSC.

### Incubation experiments

The incubation experiments were performed in 100 mL glass vials with rubber stoppers and 2 g of fresh soil (dry weight). One milliliter of carbonaceous material (biochar, AC, SWCNTs and MWCNTs) in suspension was added to the vials at various concentrations (0.004, 0.4, and 40 mg/mL) and thoroughly mixed with the soil, resulting in various carbonaceous material concentrations in the soil (0.2, 20, and 2,000 mg/kg). The suspension of the carbonaceous materials was prepared according to Zhou *et al.* (2013)^29^. A ^14^C-catechol water solution (100 μL, 356.2 kBq/mL and 13.9 μg/mL) was then added to the glass vials and thoroughly mixed with the soil-carbonaceous material matrix. The soil moisture was adjusted to 60% of the maximal water holding capacity, and the soil-carbonaceous materials matrix was thoroughly mixed again. All vials were incubated at 25 °C in a dark climate chamber for 61 days. During the incubation, the ^14^CO_2_ released from the soil was adsorbed by 1 mL of 1 M NaOH in a scintillation vial, which was suspended from the bottom of the rubber stopper. The scintillation vials containing 1 M NaOH were replaced at regular intervals, and the radioactivity of ^14^CO_2_ in the NaOH solution was measured using the LSC. All incubation trials were performed in triplicate.

### Fractionation, HP-^14^C-GPC analysis and determination of radioactive substances in soil

At the end of the incubation, soil samples were fractionated into DOM and various humic substances fractions, the molecular size distribution of the ^14^C-catechol derived residues was analyzed by high-performance radio gel permeation chromatography (HP-^14^C-GPC) and the quantification of radioactivity was performed by LSC (see Supplementary information).

### Effects of carbonaceous materials on the microbial biomass C in the soil

To evaluate the effects of carbonaceous materials on the soil microbial biomass C, 15 g of fresh soil (dry weight) was placed in 100 mL glass vials, and then, 0.75 mL of the carbonaceous material suspension at various concentrations (0.004, 0.4, and 40 mg/mL) was added to the vials and thoroughly mixed with the soil. This resulted in various carbonaceous material concentrations in the soil (0.2, 20, and 2,000 mg/kg). The soil water content was adjusted to 60% of the maximal water holding capacity, and the vials were covered with Parafilm to maintain the soil moisture throughout the incubation period. Evaporation was compensated for by adding distilled water every four days. All vials were incubated in the dark at 25 °C for 61 days. All incubation trials were performed six times.

At the end of the incubation, the microbial biomass carbon in the soil was determined using the chloroform fumigation extraction method^48^. A value of 0.45 was used for the fraction of biomass C. Half of the vials were fumigated with ethanol-free chloroform for 24 h. Both the non-fumigated and fumigated soils were extracted with 60 mL of 0.05 M K_2_SO_4_ by shaking for 1 h and then filtered. The C content in the extracts was determined using a TOC analyzer (Multi N/C 2100, Jena, Germany). The soil microbial biomass C was calculated as


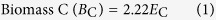


where, *E*_C_ equals the organic C extracted from fumigated soil (mg/kg) minus the organic C extracted from non-fumigated soil (mg/kg)^48^.

### Bacterial diversity analysis using 454 pyro-sequencing

Aliquot of 1 g of the moist soil samples were collected at 61 days in the incubation experiment. The microbial genomic DNA was extracted from a 0.5 g subsample of the soil using a FastDNA spin kit for soil (MP Biomedicals) based on the manufacturer’s instructions. The quality and quantity of DNA were checked using a NanoDrop spectrophotometer (NanoDrop Technologies, Wilmington, DE, USA). In the case of the AC amendment treatment, the purity and concentration of the total nucleic acid in the DNA were not sufficient and hence further analysis on the AC amendment treatment was not performed. Amplicon pyrosequencing was performed on a Roche 454 GS FLX instrument (Roche Diagnostics Corporation, Branford, CT, USA) by analyzing the V4 regions of the 16S rRNA genes as previously described by Wu *et al.* (2013)^49^. The tagged 515f and 907r primers were used to amplify the V4 region of 16S rRNA genes. Triplicate PCR amplicons per sample were pooled, purified, and combined in equimolar ratios into a single tube in preparation for the pyrosequencing analysis (see Supplementary information).

## Data analysis

The adsorption-desorption data of ^14^C-catechol on soil and carbonaceous materials were fitted to the Freundlich isotherm (Equation 2):


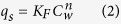


where, *q*_*s*_ (mmol/kg) and *C*_w_ (mmol/L) are the concentrations of ^14^C-catechol on the adsorbents (soil and carbonaceous materials) and in an aqueous solution at equilibrium, respectively. *K*_F_ is the Freundlich affinity coefficient (mmol^1–n^L^n^/kg), and *n* is the Freundlich linearity index (dimensionless). The data were fit to the Freundlich model using the non-linear regression function of the Sigma Plot 11.0 software.

The solid–water distribution coefficient (*K*_d_, L/kg) at various concentrations was calculated as


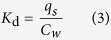


The statistical analyses were performed with the SPSS 16.0 software, and the significance level was set at *P* = 0.05. The effects of the carbonaceous material treatments on the cumulative ^14^CO_2_, ^14^C in DOM, ^14^C in the various humic substances fractions and the soil microbial biomass C were analyzed using two-way analysis of variance (ANOVA). The type of carbonaceous material (biochar, AC, SWCNTs, and MWCNTs) and the addition rate (0, 0.2, 20, and 2,000 mg/kg) were used as two independent variables. And the interactions between carbonaceous material type and addition rate were evaluated by the “test of between-subjects effects” function of two-way ANOVA analysis in the package of SPSS16.0. The significance levels and *F* values of the two-way ANOVA were obtained using the General Linear Model in SPSS 16.0.

## Additional Information

**How to cite this article**: Shan, J. *et al.* Biochar, activated carbon, and carbon nanotubes have different effects on fate of ^14^C-catechol and microbial community in soil. *Sci. Rep.* **5**, 16000; doi: 10.1038/srep16000 (2015).

## Supplementary Material

Supplementary Information

## Figures and Tables

**Table 1:** Freundlich adsorption-desorption isotherm parameters of ^14^ C-catechol on soil and carbonaceous adsorbents (Biochar, Activated carbon, SWCNTs, and MWCNTs).

Adsorbents	Adsorption					Desorption				
	log *K*_F_ (mmol^1-n^ L^n^/kg)	*n*	*R*^*2*^	log *K*_d_		log *K*_F_(mmol^1-n^ L^n^/kg)	*n*	*R*^*2*^	log *K*_d_	
				0.01 mmol/L	0.1 mmol/L				0.01 mmol/L	0.1 mmol/L
Soil	2.55 ± 0.07	0.67 ± 0.01	0.99	3.22	2.89	3.19 ± 0.06	0.74 ± 0.02	0.99	3.71	3.45
Biochar	4.13 ± 0.01	0.92 ± 0.01	>0.99	4.28	4.20	4.51 ± 0.02	0.92 ± 0.02	> 0.99	4.66	4.59
Activated carbon	4.28 ± 0.08	0.62 ± 0.04	0.98	5.05	4.67	4.68 ± 0.03	0.91 ± 0.01	> 0.99	4.86	4.77
SWCNTs	4.99 ± 0.12	0.59 ± 0.02	0.99	5.66	5.32	5.45 ± 0.10	0.71 ± 0.03	0.99	6.02	5.74
MWCNTs	4.61 ± 0.04	0.71 ± 0.01	>0.99	5.28	4.95	5.06 ± 0.04	0.75 ± 0.01	> 0.99	5.55	5.30

**Table 2:** Distribution and recovery of radioactivity from ^14^ C-catechol in soil with and without (Control soil) different amounts of carbonaceous materials (Biochar, Activated carbon, SWCNTs, and MWCNTs) after 61 days of incubation at 25 °C.

Treatment	Carbonaceous material concentration (mg/kg)	% of initially applied ^14^C						
		^14^CO_2_	DOM	Fulvic acids	Humic acids	Soluble humin	Insoluble humin	Recovery
Control soil	0	18.48 ± 0.85	0.26 ± 0.06	23.88 ± 0.64	18.19 ± 1.04	16.26 ± 0.88	17.54 ± 1.08	94.61 ± 2.12
Biochar	0.2	16.65 ± 0.54	0.32 ± 0.04	25.15 ± 0.12	17.51 ± 0.77	16.71 ± 0.47	17.05 ± 0.28	93.39 ± 1.80
	20	18.17 ± 1.18	0.30 ± 0.04	23.99 ± 0.94	18.00 ± 0.30	15.62 ± 0.82	17.66 ± 0.44	93.74 ± 0.71
	2000	17.72 ± 1.42	0.46 ± 0.11	24.05 ± 0.59	17.64 ± 1.63	15.52 ± 0.68	15.54 ± 2.14	90.93 ± 1.62
Activated carbon	0.2	15.37 ± 0.33	0.29 ± 0.02	24.65 ± 1.36	18.53 ± 1.74	15.95 ± 0.28	17.24 ± 0.69	92.04 ± 1.73
	20	15.43 ± 1.07	0.28 ± 0.03	24.93 ± 0.48	19.55 ± 0.65	16.29 ± 0.78	17.78 ± 0.77	94.27 ± 1.26
	2000	15.21 ± 0.67	0.20 ± 0.03	24.32 ± 0.48	17.38 ± 0.21	17.43 ± 0.55	20.44 ± 0.92	94.97 ± 0.63
SWCNTs	0.2	19.56 ± 0.43	0.24 ± 0.03	21.97 ± 3.18	20.01 ± 3.18	15.76 ± 0.29	17.74 ± 0.54	95.28 ± 1.01
	20	18.41 ± 0.05	0.20 ± 0.02	23.14 ± 0.84	17.99 ± 1.10	16.06 ± 0.37	17.35 ± 2.40	93.15 ± 1.52
	2000	14.94 ± 0.56	0.70 ± 0.37	25.84 ± 0.69	16.95 ± 1.83	17.32 ± 0.69	19.77 ± 0.50	95.52 ± 1.65
MWCNTs	0.2	22.00 ± 1.24	0.26 ± 0.02	23.55 ± 0.19	16.89 ± 1.28	14.89 ± 0.86	17.59 ± 0.85	95.18 ± 1.82
	20	19.01 ± 0.42	0.28 ± 0.03	23.94 ± 0.23	16.58 ± 1.42	14.64 ± 0.44	18.54 ± 0.69	93.00 ± 1.95
	2000	20.03 ± 1.44	0.28 ± 0.02	22.53 ± 0.23	15.50 ± 0.29	14.99 ± 0.30	19.12 ± 4.06	92.45 ± 3.76

The residual radioactivity was fractionated into dissolved organic matter (DOM), fulvic acids, humic acids, soluble humin, and insoluble humin fractions. The values for the control soil treatment are averages with a standard deviation calculated from twelve individual experiments, whereas all other values are averages with a standard deviation derived from three separate experiments.
