# Supplementary material for: Biochar, activated carbon, and carbon nanotubes have different effects on fate of 14C-catechol and microbial community in soil
Source: Sci Rep. 2015 Oct 30;5:16000. doi: 10.1038/srep16000 (PMC4626844; doi:10.1038/srep16000)
Supplement: Supplementary Information [file srep16000-s1.doc]

Supplementary information for

# Biochar, activated carbon, and carbon nanotubes have different effects on fate of ^14^C-catechol and microbial community in soil

# Authors: Jun Shan^1,2^, Rong Ji^3^, Yongjie Yu^4^, Zubin Xie^1^, Xiaoyuan Yan^1,2^

Address: ^1^ State Key Laboratory of Soil and Sustainable Agriculture, Institute of Soil Science, Chinese Academy of Sciences, Nanjing 210008, China

^2^ Changshu Agro-ecological Experimental Station, Chinese Academy of Sciences, Changshu 215555, China

^3^ State Key Laboratory of Pollution Control and Resource Reuse, School of the Environment, Nanjing University, 163 Xianlin Avenue, Nanjing 210023, China

^4^ College of Applied Meteorology, Nanjing University of Information Science and Technology, Nanjing 210044, China

*: Corresponding Author email: [yanxy@issas.ac.cn](mailto:yanxy@issas.ac.cn); Tel: +86-025 8688 1530 Fax: +86-025 8688 1000

Numbers of pages: 7

Numbers of figures: 2

Numbers of tables: 1

**Methods**

**Fractionation of radioactive substances in soil**

After 61 days of incubation, an aliquot of 1 g soil samples (dry weight) was sequentially extracted with distilled water for DOM and with 0.1 M anoxic NaOH for the FA, HA and humin fractions, based on their alkaline solubility according to the method of Shan et al. (2010)^1^. An aliquot (6 mL) of each alkaline extract containing fulvic and humic acids was freeze-dried and re-dissolved again in 0.1 M anoxic NaOH to analyze the molecular size distribution of radioactive residues among the humic substances. High-performance radio gel permeation chromatography (HP-^14^C-GPC) was used for the analysis (see below). An aliquot of 1 g of the humin fraction was then further silylated according to the method derived by Butenschoen et al. (2009)^2^, in which the radioactive residues associated with humin by physico-chemical interactions or by covalent bonds were distinguished. The radioactive residues associated with humin by physico-chemical interactions were defined as the soluble humin fractions, whereas the radioactive residues chemically bound to humin through covalent bonds were defined as the insoluble humin fractions.

**HP-^14^C-GPC analysis and determination of the radioactivity**

Prior to the HP-^14^C-GPC analysis, the alkaline suspension was further centrifuged for 30 min at 20,000 *g*/10 ºC, and an aliquot of the supernatant (100 μL) was extracted for analysis. Detailed information about the molecular size distribution analysis can be found in Shan et al. (2014)^3^.

Radioactivity determination was conducted in a LSC (LS 6500; Beckman Coulter; USA). For the determination of ^14^C in the water supernatant in the adsorption-desorption experiments, 0.5 mL of the supernatant was mixed with a 2 mL scintillation cocktail (Gold Star multipurpose; Meridian Biotechnologies Ltd. UK). For ^14^CO_2_ in 1 M NaOH solution, 1 mL of a NaOH solution was mixed with the 2 mL scintillation cocktail. For water extracts and alkaline extracts, 1 and 0.1 mL extracts were mixed with 2 and 15 mL of a scintillation cocktail, respectively. A certain quantity of the solid samples (the humin and insoluble humin fractions) was first oxidized by a biological oxidizer (OX500; Zinsser Analytic GmbH, Germany). The resulting ^14^CO_2_ was absorbed by a 15 mL Oxysolve C-400 cocktail and then counted using a LSC.

**Pyrosequencing data processing**

The 454 pyrosequencing data processing was conducted using Mothur software v1.30.2^4^ for taxonomic identification. The sequences were quality trimmed (>30 quality score and 300 bp in length), and were aligned against the SILVA bacterial 16SrRNA gene databases using the Needleman algorithm. Chimeric sequences were identified and removed using Chimera-uchime^5^. The high-quality bacterial sequences were extracted and used to generate a distance matrix and clustering with the average neighbor algorithm. Representative sequences for each operational taxonomic unit (OTU) using a 97% sequence identity were obtained for further analysis. The closest isolates to the representative sequence of each OTU were retrieved from GenBank using the BLAST search engine (<http://blast.ncbi.nlm.nih.gov/Blast.cgi>). Principal coordinate analysis (PCoA) was performed based on the Bray-Curtis dissimilarity distances between the libraries with Mothur to evaluate the differences in the overall community composition among the various soil samples.

Table S1: Selected physico-chemical properties of the carbonaceous materials used in this study.

| Carbonaceous materials | Surface area (m^2^/g) | Average pore width (Å ) | Element compositions (w/w %) | | | | |
| --- | --- | --- | --- | --- | --- | --- | --- |
|  |  |  | N | C | H | S | O |
| Biochar | 55.5 | 5.4 | 0.24 | 75.21 | 0.47 | 15.09 | 9.0 |
| Activated carbon | 83.6 | 4.8 | 0.26 | 59.17 | 1.27 | 9.05 | 30.3 |
| SWCNTs | 310.3 | 167.0 | 0.22 | 91.08 | 0.57 | 3.27 | 4.9 |
| MWCNTs | 157.3 | 991.6 | 0.20 | 97.62 | 0.53 | 2.35 | － |


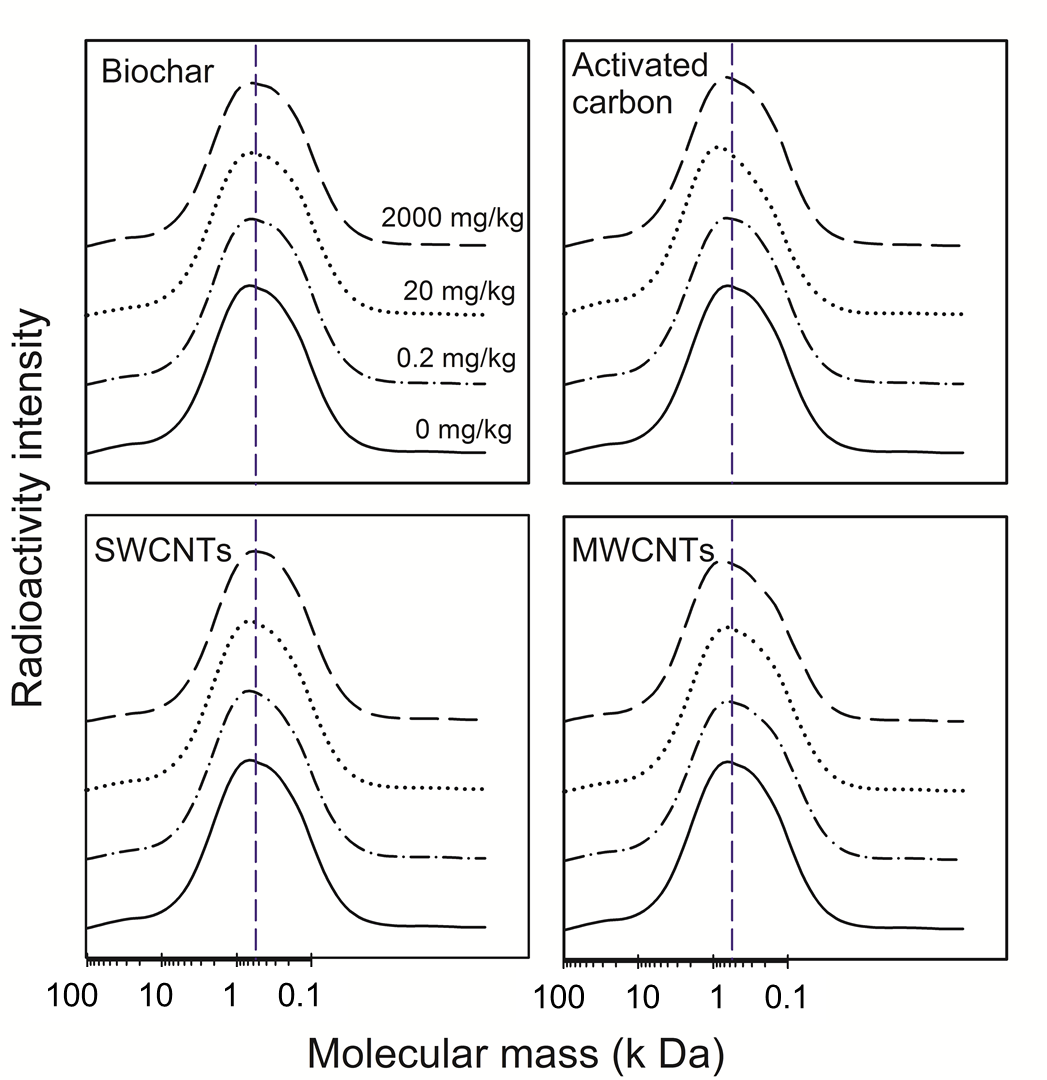


Figure S1: Molecular size distribution of ^14^C-catechol derived residues within the alkaline extractable soil humic substances after 61 days of incubation in soil in the absence of carbonaceous materials (0 mg/kg) and in the presence of various concentrations (0.2, 20, and 2,000 mg/kg) of carbonaceous materials. The radioactivity chromatograms were normalized by area to facilitate the comparison.


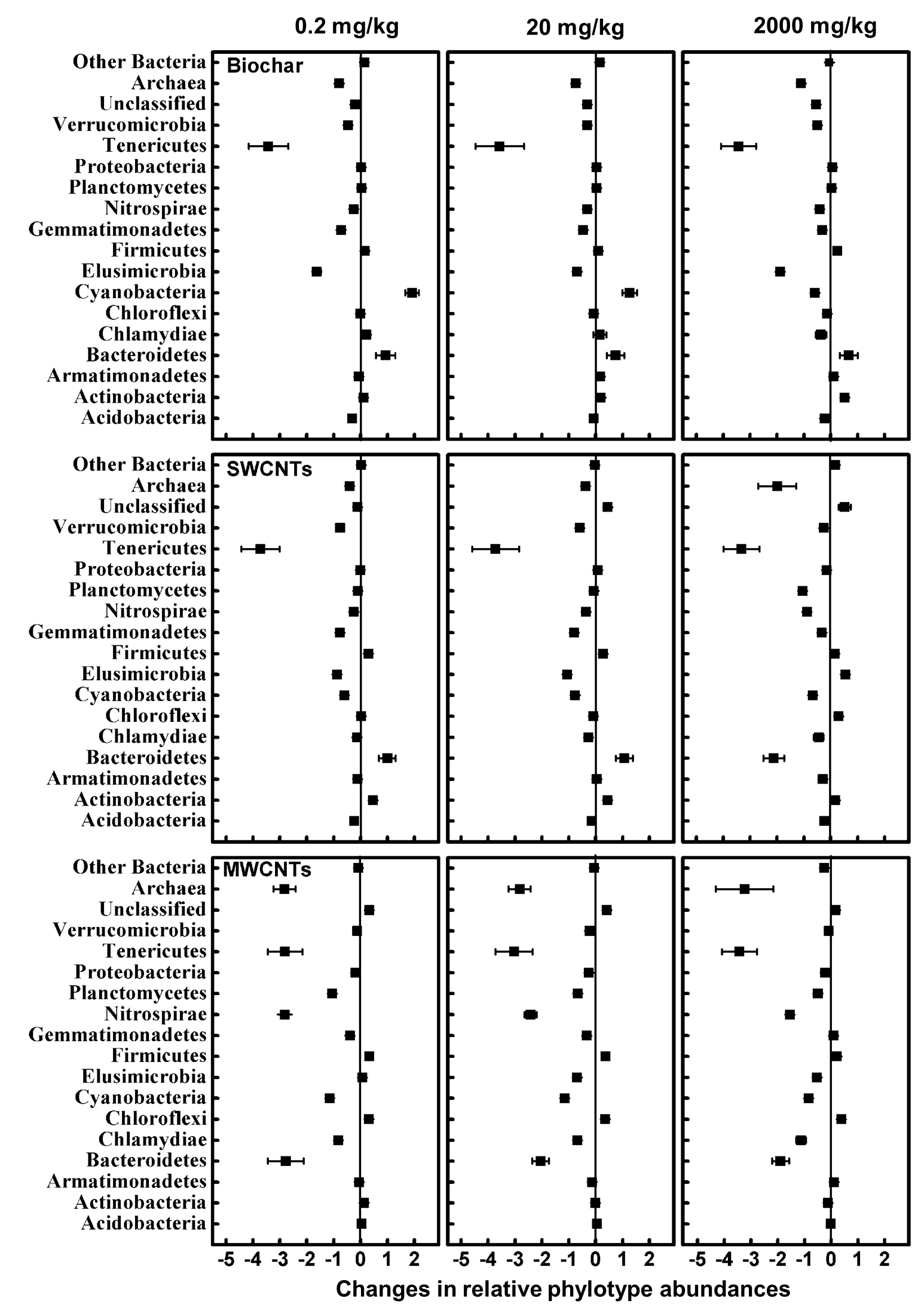


Figure S2. Percentage change (response ratio) of phylotype abundance in soil amended with various concentrations of carbonaceous materials (0.2, 20, and 2,000 mg/kg) relative to those for the control soil. The means for the samples with carbonaceous materials treatments were significantly different (*P* < 0.05) from that of the control treatment if the horizontal error bar did not overlap the zero line.

**References**

1. Shan, J. et al. Effects of biochar and the geophagous earthworm *Metaphire guillelmi* on fate of ^14^C-catechol in an agricultural soil. *Chemosphere* **107**, 109-114 (2014).

2. Butenschoen, O., Ji, R., Schäffer, A. & Scheu, S. The fate of catechol in soil as affected by earthworms and clay. *Soil Biol. Biochem.* **41**, 330-339 (2009).

3. Shan, J., Brune, A. & Ji, R. Selective digestion of the proteinaceous component of humic substances by the geophagous earthworms *Metaphire guillelmi* and *Amynthas corrugatus*. *Soil Biol. Biochem.* **42**, 1455-1462 (2010).

4. Schloss P, et al. Introducing mothur: open-source, platform-independent, community-supported software for describing and comparing microbial communities. *Appl. Environ. Microbiol.* **75**, 7537-7541 (2009).

5. Edgar R, Haas B, Clemente J, Quince C, Knight R. UCHIME improves sensitivity and speed of chimera detection. *Bioinformatics* **27**, 2194-2200 (2011).
